# Novel polymorphisms in *TICAM2* and *NOD1* associated with tuberculosis progression phenotypes in Ethiopian populations

**DOI:** 10.1017/gheg.2017.17

**Published:** 2018-01-23

**Authors:** E. Mekonnen, E. Bekele, C. M. Stein

**Affiliations:** 1 Microbial, Cellular, Molecular Biology Department, Addis Ababa University, P.O.Box:17087, Addis Ababa, Ethiopia; 2 Health Biotechnology Department, Institute of Biotechnology, Addis Ababa University, P.O.Box:17087, Addis Ababa, Ethiopia; 3 Microbial, Cellular, Molecular Biology Department, Addis Ababa University, Ethiopia; 4 Department of Population & Quantitative Health Sciences, Center for Proteomics & Bioinformatics, and Tuberculosis Research Unit, Case Western Reserve University, USA

**Keywords:** Case-contact study, genetic epidemiology, human genetic polymorphism, innate immunity, latent tuberculosis infection, tuberculosis genetics

## Abstract

**Background:**

Infection by *Mycobacterium tuberculosis* (*Mtb*) is a necessary but not sufficient cause for tuberculosis (TB). Although numerous studies suggest human genetic variation may influence TB pathogenesis, there is a conspicuous lack of replication, likely due to imprecise phenotype definition. We aimed to replicate novel findings from a Ugandan cohort in Ethiopian populations.

**Method:**

We ascertained TB cases and household controls (*n* = 292) from three different ethnic groups. Latent *Mtb* infection was determined using Quantiferon to develop reliable TB progression phenotypes. We sequenced exonic regions of *TICAM2* and *NOD1*.

**Result:**

Significant novel associations were observed between two variants in *NOD1* and TB: rs751770147 [unadjusted *p* = 7.28 × 10^−5^] and chr7:30477156(T), a novel variant, [unadjusted *p* = 1.04 × 10^−4^]. Two SNPs in *TICAM2* were nominally associated with TB, including rs2288384 [unadjusted *p* = 0.003]. Haplotype-based association tests supported the SNP-based results.

**Conclusion:**

We replicated the association of *TICAM2* and *NOD1* with TB and identified novel genetic associations with TB in Ethiopian populations.

## Introduction

Tuberculosis (TB), caused by *Mycobacterium tuberculosis* (*Mtb*), is a major public health threat globally. It is estimated that one-third of the world is infected with *Mtb*, and roughly 10% of these individuals progress to active TB disease. TB pathogenesis follows a two-stage process: first, uninfected individuals become exposed to infectious TB cases, and may progress to *Mtb* infection, though not all subjects exposed to TB become infected [1]. In the second stage, ~10% of *Mtb*-infected individuals progress to active TB disease. These two stages may have different genetic underpinnings. In 2015, 10.4 million developed active TB and another 1.8 million died from the disease (http://www.who.int/mediacentre/factsheets/fs104/en/). Ethiopia is one of the high TB burden countries. In 2014, the estimated prevalence of TB in Ethiopia was 200 per 100 000 and incidence of 207 per 100 000 [2].

Although TB is primarily a pulmonary disease (PTB) that is initiated by the deposition of *Mtb* contained in aerosol droplets onto lung alveolar surfaces, it is also characterized by a variable course of disease in terms of latency, reactivation and progression to active disease; variation in affected sites; and, variation in severity and mortality that can all be modulated by nutrition, age, sex and genetics [3]. Although *Mtb* is a necessary bacterial cause, it is not sufficient to cause the disease. In other words, active TB disease is not exactly an inevitable outcome of exposure to, or infection by, *Mtb*. Both innate and adaptive immunity mechanisms are involved in determining the outcome of *Mtb* infection. In this respect, different genetic polymorphisms have been found which are associated with increased susceptibility to and severity of TB. Some of these polymorphisms are functional, but for many of these no functional (immunologic) changes have been demonstrated yet, and these associations need further confirmation [4]. In addition, pathogenic diversity may play a role in disease progression disparity since some *Mtb* strains are reportedly more virulent than others, as defined by increased transmissibility, rapid pathogenesis, higher morbidity and mortality in infected individuals [5, 6]. However, results of genetic association studies have varied widely [7], likely due to complex multi-factorial nature of TB that impacts our ability to diagnose and characterize the disease phenotype in a manner that is amenable for genetic analysis. These difficulties translate into major gaps in our knowledge and, therefore, a comprehensive genetic model for TB has not yet emerged [8, 9]. The genetic complexity of TB is understandable since immune responses to *Mtb* infection, molecular signaling patterns and the extent of tissue involvement change over the course of the disease, and it is unlikely that any single factor can adequately explain susceptibility to TB. Thus, numerous genetic variants have been variably associated with susceptibility to TB [10, 11]. Despite these limitations after numerous twin, candidate-gene, as well as genome-wide linkage and association studies in different populations, the evidence for a human genetic component in predisposition to TB susceptibility is considered incontrovertible [12, 13].

In Africa, where both high human genetic variation and TB disease are still prevalent, genetic epidemiological investigations are crucial and the discovery of genetic associations with strong effect sizes that impact susceptibility/resistance to disease could guide public health policy [11, 14]. Several genetic association studies of TB have been carried out in Africa which reported positive, suggestive or no association results [10, 11]. For example, using the complementary approach of candidate gene analysis, case-control studies of West African samples have identified associations with variants in several genes, including *SLC11A1* [15, 16]; *IL1B* [17]; vitamin D receptor [18, 19]; *CD209 (DC-SIGN), PTX3* [20]; and *P2X7* genes [21]. In East Africa, a combined linkage and association study of Ugandans has shown that IL10, interferon gamma receptor 1 (*IFNGR1*), and TNF alpha receptor 1 (*TNFR1*) variants are linked to active TB, but not with latent infection [8]. And, in the first genome-wide linkage scan for a major infectious disease in Africans, significant evidence of linkage was found on chromosomes Xq27 and 15q11 [22], further supporting the conclusion that TB susceptibility loci exist. A recent study in Ugandan populations based on tagSNPs that mostly fall in intronic regions of immunity genes identified *TICAM2* and *NOD1* as candidate genes among others [23]. There is only one previously published study that investigated genetic susceptibility to TB in Ethiopian populations [24]. The authors investigated polymorphisms in the *SFTPA1* and *SFTPA2* genes, both expressed in the lungs, and identified four polymorphisms that modify the risk of TB susceptibility.

In the present study, we focused on two innate immunity genes, *NOD1* [Nucleotide-binding Oligmerization Domain containing 1] and *TICAM2* [Toll/Interleukin-1 Receptor Domain-Containing Adaptor Molecule 2], based on a recent finding [23] in an East African population that indicated significant statistical association and another study demonstrating a biological plausibility of *TICAM2-NOD1* synergistic action [25] and, thus, were deemed to warrant an effort to replicate those findings in an independent population. *TICAM2* is a member of the pattern-recognition-receptors called Toll-like-receptors (TLRs) that sense pathogen-associated-molecular-patterns in the extracellular environment as well as in endosomes. TLRs are primarily expressed in immune cells, such as macrophages, monocytes, B-lymphocytes, and dendritic cells. *TICAM2*, also known as *TRAM*, mediates innate immune responses through extracellular recognition of microbes and by facilitating the expression of IFN-inducible genes [26–28]. Its physical structure and interaction properties have been elucidated recently [29]. In addition to the recent genetic association finding with TB [23], it was also shown to be associated with a candidate TB vaccine trial outcome [30]. *NOD1* is a member of the intracellular pathogen-recognition-receptors, nod-like receptors (NLRs), which are important for the recognition of unique muropeptides of bacterial peptidoglycan fragments (derived predominantly from the cell walls of *Mtb*) or damage-associated-molecular-patterns that localize to the cytosol. Following microbial sensing, *NOD1* directly recruits factors which initiate intracellular signaling cascade that leads to the activation of transcriptional responses culminating in the expression of a subset of innate immunity genes involved in inflammation (e.g., production of proinflammatory cytokines and chemokines), antimicrobial mechanisms and autophagy in cells responsible for eliminating *Mtb* including epithelial cells, alveolar macrophages and monocyte-derived macrophages [31]. Mutations in several NLR members were found to be associated with the development of inflammatory disorders [32–34] including TB [23].

There are several reasons for regarding Ethiopia as a ‘model human population’ for the study of the genetic profile of virtually any phenotype of interest. Several anthropologic, linguistic, and genetic studies have suggested that humans originated in the African continent [35, 36] with the range and weight of evidence for the Ethiopian source predominating any other single alternative in Africa [37–41]. In this regard, we have recently demonstrated using genome-wide data the existence of not only unparalleled overall human genetic diversity among present-day Ethiopian populations, but also that Ethiopians possess the greatest proportion of novel variation most of which are unshared (private) and rare [42]. This finding can be explained away, in combination or alone, by the effects of neither genetic drift, population bottleneck, migration/admixture events, nor the extent and pattern of LD decay; but more logically by the longer mutational time-scale needed to generate the extent of observed diversity, and thus, the much older population history of present-day Ethiopians. These and other evidence posit Ethiopia within Africa as the most probable single source of modern humans. Therefore, it is reasonable to propose that the tremendous genetic diversity existing in Ethiopia can be harnessed towards the understanding of the genetic basis of various infectious and non-infectious diseases that afflict humanity. With respect to the current study of human genetic susceptibility to TB, Ethiopia is also ‘well situated’. There is increasing evidence indicating the common origin in Ethiopia of MTBC (mycobacterium-tuberculosis-complex) and humans and that MTBC has been co-evolving with anatomically modern humans. These conclusions are based on the observed congruence in their phylogeographies, the dating of major branching events, and the discovery of a unique lineage of MTBC localized only in Ethiopia [43]. Therefore, studies of the impact of *Mtb*–human interactions would best be investigated in an Ethiopian setting.

The primary hypothesis of the present study was that the reasons for non-replication of TB genetic association results may be explained by the lack of precise definition of TB phenotypes that frustrates the discovery of underlying gene-disease associations or lead to spurious conclusions. Clearly defined traits can help increase power to detect disease-predisposing loci and thus more informative than studying a heterogeneous lump of a complex phenotype. This study focused on finding a method of unraveling TB phenotype complexity by drawing a clear definition of the TB trait based on its known natural progression stages from exposure, to infection, and progression to active disease. This ensures that intermediate stages of the disease are included as phenotypes of interest, since they may represent distinct TB phenotypes with respective immuno-genetic profiles [44–46]. The recent identification of *TICAM2* and *NOD1* association with TB in Ugandan populations was made possible by a similar methodology [23]. It was also hypothesized that the Ethiopian setting could indirectly help to explore the possibility that *Mtb*-human co-evolution may result in differential TB genetic risk profiles across population categories. There is accumulated evidence indicating that (A) *Mtb* strain diversity is not only non-random across the globe but also associated with specific TB phenotypes, and (B) the existence of high *Mtb* strain diversity in Ethiopia including a unique strain [47–49]. Accordingly, the current study will help to assess whether recent findings in an East African population, Uganda, based on similar methods of defining TB phenotypes replicate in an independent neighboring Ethiopian population and assess if there are common signatures of population-specific association with TB phenotypes.

## Methods

### Ethical considerations

The research proposal received Ethical Clearance from the relevant institutions in Ethiopia: the Ethical Review Committee of the Department of Biology at Addis Ababa University and the National Health Research Ethics Review Committee of the Federal Ministry of Science and Technology of Ethiopia (certificate#: RDHE/37-92/2010). Samples were collected after obtaining informed and signed consent.

### Phenotyping (clinical characterization)

Cases of active PTB were diagnosed by hospital medical personnel as per the guidelines provided by the Federal Ministry of Health of Ethiopia [50] and all cases were undergoing treatment during recruitment. In Ethiopia the diagnosis of TB is based on the recommendations of the National TB and Leprosy Control Programme (NTLC) [51] as follows:

- Patients presenting with symptoms suggestive of PTB who had productive cough for 3 weeks or more with at least two positive sputum smears or one positive smear and X-ray findings consistent with active PTB are classified as smear-positive PTB cases.
- Patients presenting with cough of 3 weeks or more with initial three negative smears and no clinical response to a course of broad-spectrum antibiotics, three negative smear results after a course of broad-spectrum antibiotics, X-ray findings consistent with active PTB and decided by a clinician to be treated with anti-TB chemotherapy are classified as smear-negative PTB cases.
- Patients presenting with dry cough of 3 weeks or more are diagnosed based on strong clinical evidence and X-ray findings consistent with active TB.

Other diagnostic services such as Mycobacterial cultures and pathological services are not available for routine purposes and as a result, the NTLC advocates adherence to the above algorithm [52].

For each PTB case, all available and consenting unrelated household contacts, mostly spouses, were selected as controls. This ensured that exposure to TB could be known with certainty, thus reducing the potential for misclassification of controls [7]. The QuantiFERON^®^-TB Gold In-Tube (QFT^®^) kit was employed to test samples of asymptomatic household contacts of active TB patients for latent infection. This is an *in vitro* diagnostic method that specifically tests for latent *Mtb* infection using peptide antigens that are absent from all BCG strains and from most non-tuberculosis mycobacteria. The IFN-gamma level of the Nil tube is compared with the TB antigen and Mitogen tubes to determine infection status (Supplementary Table S1). All subjects were tested for HIV, and HIV-positive subjects were excluded from further analyses.

**Fig. 1.**
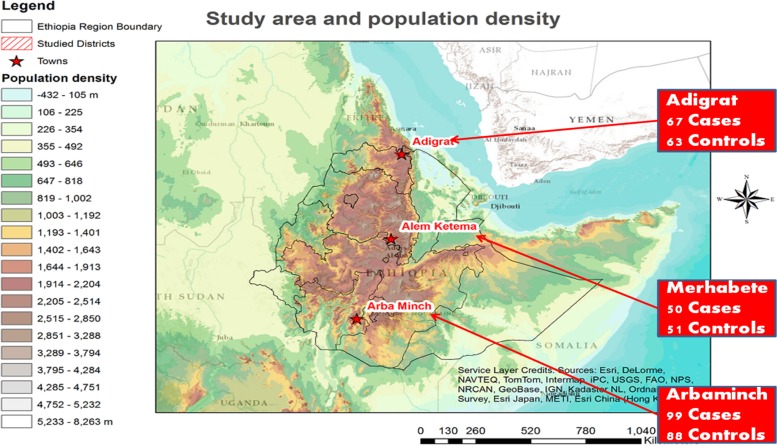
The sample-populations were selected in a manner that traverses some of the major ethno-linguistic-geographic groups in Ethiopia: Tigray, Amhara, and Gamo.

In this study, samples were taken from three regions representing some of the North, Central and Southern ethno-geographic categories (EGCs) of Ethiopia: Merhabet (Amhara ethnic group, Amharic speakers), Adigrat (Tigray ethnic group, Tigringna speakers), and Arbaminch (Gamo ethnic group, Gamingna speakers), respectively. The Amhara and Tigray populations have a Semetic background while the Gamo is Omotic (Fig. 1).

### Genotyping/exome sequencing

Genomic DNA was extracted from buffy coat samples using QIAGENE (FlexiGene) as per product instructions. DNA quality and quantity were determined using both Nanodrop and Qubit. Exonic regions of candidate genes were sequenced, alligned and variants called using Illumina MiSeq and the Homosapiens/UCSC/hg19 human genome reference panel at the Genome Sequencing Core Facility of Case Western Reserve University (Supplemental Table S2). Samples were randomly placed on sequencing plates so that there was no bias by case/control status or by ethnicity.

### DNA sequence data quality control (QC)

QC thresholds were selected that maximized the number of individuals and markers/SNPs included while ensuring appropriate QC for both. For the per-individual QC, individuals with less than 90% genotyping rate (i.e., individuals missing genotypes for more than 10% of the total markers) were removed. In the per-marker QC, markers with genotyping failure rate of less than 95% (i.e., markers genotyped in less than 95% of all samples), markers which failed Hardy–Weinberg–Equilibrium (HWE) deviation test with *p* < 0.001, and those with minor allele frequency (MAF) less than 0.01 were all removed. Tests for significant bias in genotyping rate between cases and controls were all non-significant. Coverage for these specific exonic regions was 100%. The total number of exonic nucleotide sequences was 11 927 (*TICAM2* = 3578, *NOD1* = 8349) with an average genotyping rate of 0.86–0.92. Most of the filtered out markers (91–95%) were discarded because they were monomorphic and thus uninformative for downstream association analysis (Supplementary Table S3).

### Genetic association analysis

Unrelated case-control phenotype categories were developed in an increasingly restrictive or exclusive manner. This set up helps to study the impact of phenotype definition and perform a sensitivity analysis by observing the trend of association test statistic across the phenotype classifications and is one of the major strengths of this study. First, all controls were ascertained by virtue of their close association with active pulmonary TB patients as household contacts/care-givers and, therefore, most likely to have been exposed to *Mtb*. Second, they have been living in generally TB-endemic communities under conditions conducive to TB exposure and transmission (e.g., housing and population density). Third, controls were deemed negative for symptoms of active TB after a physical examination by qualified clinicians thus forming the group ‘No Active TB’ meaning ‘no symptoms of active TB after *Mtb* exposure’. Fourth, IGRA was performed to ensure whether the infection has been established or not after exposure, i.e., discriminate between the phenotypes of ‘LTBI: susceptibility/progression to infection’ and ‘No LTBI: resistance to infection’, respectively. [LTBI: Latent-TB-Infection].

We constructed two primary case *v.* control test-models to examine whether these genetic variants predispose to TB disease or *Mtb* infection, respectively:

- Test-model 1: ‘Active TB’ *v.* ‘No Active TB’ (153 cases, 139 controls)
- Test-model 2: ‘LTBI’ *v.* ‘No LTBI’ (70 cases, 64 controls)

To avoid potential concerns with heterogeneity in the control group in test model 1, we conducted sensitivity analyses by constructing additional test models as follows:

- Test-model 3: ‘Active TB’ *v.* ‘No LTBI’ (153 cases, 64 controls)
- Test-model 4: ‘Active TB’ *v.* ‘LTBI’ (153 cases, 70 controls)

Several complementary analysis strategies were used to examine the association between the genetic variants in *NOD1* and *TICAM2* and TB phenotypes using the test models described above. All analyses were conducted using PLINK [53]. First, we compared the distribution of the minor allele in cases and controls using Pearson's χ^2^ and Fisher's exact test. We corrected for multiple testing using a Bonferroni correction: a total of 94 variants were examined between this study and another one, resulting in a corrected significance level of *α* = 0.05/94 = 5.32E-04. Correction for multiple testing was also done based on the false discovery rate (FDR) estimation algorithm of Benjamini, Hochberg and Yekutieli as implemented in PLINK. We also coded the genotypes according to dominant, additive, and recessive models to examine mode of inheritance; we found that the additive model was always the most significant (data not shown) so we proceeded with this model for further analysis. Allelic association tests were also conducted stratified by ethnic group as well as in the overall study population. Then, we conducted a logistic regression analysis, using additive coding of the SNP genotypes with the Cochrane–Armitage test assuming that each extra copy of the ‘risk’ allele increases risk equally, and included sex, age, and ethnicity as covariates in this model. To test for potential population stratification, we compared allele frequencies among the ethnic groups using a χ^2^ test, and also used multidimensional scaling (MDS) to empirically visualize and assess population stratification based on both self-declared ethnicity (EGC) of subjects and identity-by-state (IBS) of SNPs. PLINK employs complete linkage agglomerative clustering, based on pair-wise SNP IBS distance.

**Fig. 2.**
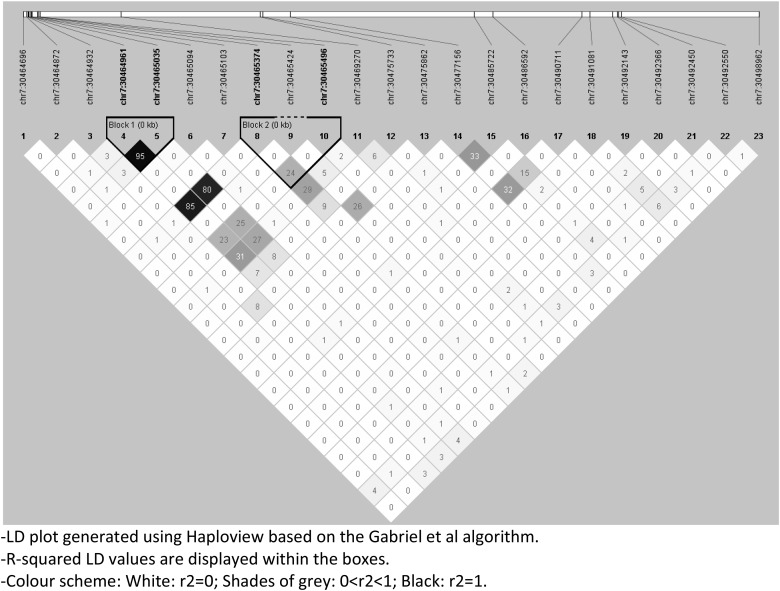
LD pattern for *NOD1*: Pair-wise *r*^2^ are plotted for *NOD1* based on 23 QC-passed SNPs in 292 individuals included in Active TB *v.* No Active TB test-model.

**Fig. 3.**
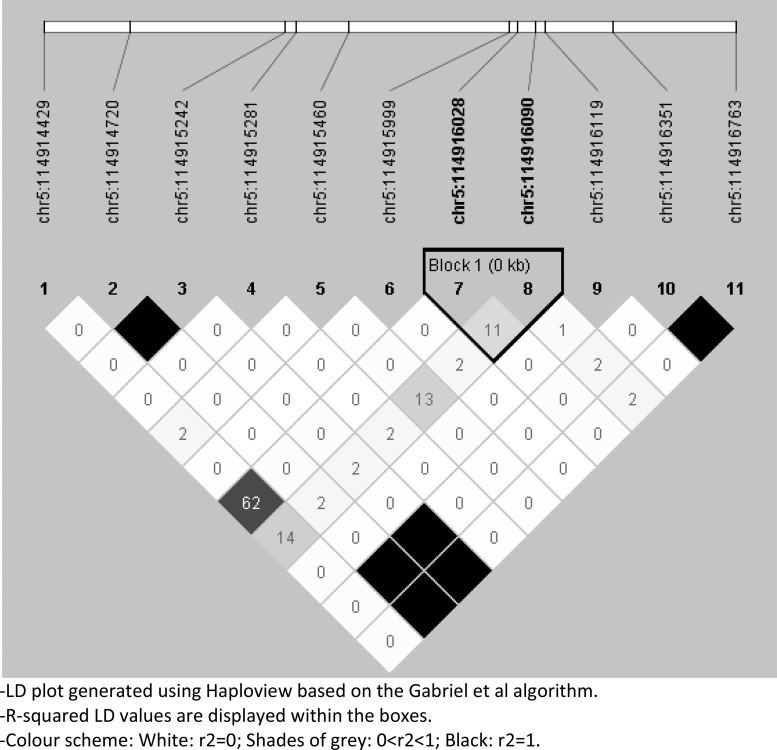
LD pattern for *TICAM2*: Pair-wise *r*^2^ are plotted for *TICAM2* based on 11 QC-passed SNPs in 292 individuals included in Active TB *v.* No Active TB test-model.

Linkage-disequilibrium patterns within the twogenes were plotted using Haploview's ‘Gabriel *et al*. algorithm’ based on QC-passed SNPs in 292 individuals included in the largest primary test-model: Active TB *v.* No Active TB (Figs 2 and 3 for NOD1 and TICAM2, respectively). Haplotype association analysis for both an omnibus and haplotype-specific tests were performed that compared the distribution of haplotypes in cases *v.* controls.

## Results

In this study, 153 TB cases and 134 contacts without TB were enrolled (Table 1). Among the non-TB cases, 70 were QFT positive and 64 were QFT negative. The QFT negative cases were more likely to be female and younger. Most of the TB cases came from the Arbaminich (Gamo) ethnicity.

**Table 1.** Demographic characteristics of participants

Descriptives	Site (ethnic group)	Subject status		
		Active TB	QFT positive	QFT negative
Sample size (N)	Merhabete (Amhara)	31	16	21
	Adigrat (Tigray)	38	22	23
	Arbaminch (Gamo)	84	32	20
	Combined population	153	70	64
% Male	Merhabete (Amhara)	58.1	76.2	62.5
	Adigrat (Tigray)	71.1	47.8	27.3
	Arbaminch (Gamo)	83.3	90.0	84.4
	Combined population	75.2	70.3	61.4
Mean age (range)	Merhabete (Amhara)	39.7 (15–70)	30.9 (23–57)	29.9 (22–55)
	Adigrat (Tigray)	38.5 (18–85)	40.3 (21–60)	27.0 (15–67)
	Arbaminch (Gamo)	30.6 (15–82)	33.3 (15–85)	32.9 (17–105)
	Combined population	34.4 (15–85)	35.0 (15–85)	29.8 (15–105)

We identified one novel variant in *TICAM2* and two novel variants in *NOD1*. Results of SNP association tests by each phenotype contrast are shown in Table 2. Detailed results for the primary test-models, as well as for the sensitivity analyses, are presented in Supplementary Tables S4 and S5. Only additive coded models attained statistical significance. Two results in *NOD1* achieved statistical significance after multiple-test correction: rs751770147 (*p* = 7.28E-05) and chr7:30477156(T) (*p* = 1.0E-04), a novel variant. These results were attained in the comparison of active TB cases to non-TB controls (test model 1). Nominally significant association results for these same two variants were also seen in sensitivity analyses for the contrast of Active TB to no LTBI, and also Active TB to LTBI (Supplementary Tables S4 and S5), providing support that these variants are associated with increased risk for TB. One of these variants, rs151170709, is a missense variant, with *p* < 0.05. Two other variants in *NOD1* showed similar trends with increased risk for TB, and four variants in *NOD1* also showed protective effects, though these were not significant after correction for multiple testing. In addition, two variants in *TICAM2* were nominally associated with increased risk for TB as seen by at least two of the contrasts, and another two variants were associated with protection against TB in two models, though these results did not attain statistical significance after multiple testing corrections. One of these variants was a novel variant. However, it is to be noted that *p* < 0.05 is sufficient for declaring replication. Only one variant, in *NOD1*, was nominally associated with risk of LTBI compared with no LTBI. Minor allele frequencies for phenotype-associated SNPs are provided in Table 3.

**Table 2.** Summary results of SNP-based association tests: primary analysis

Patterns of SNP-phenotype associations across test models: best unadjusted *p*-values							
SNPs associated with increased TB risk (OR > 1)	Candidate genes						
	NOD1					TICAM2	
	chr7:30477156(T) NOVEL VARIANT	rs751770147(T)	rs543650951(T)	rs151170709(A)	rs17159043(T)	rs256996(A)	chr5:114915999(A) NOVEL VARIANT
Primary test-model 1 Active TB *v.* No Active TB (6 SNPs)	0.000104	0.000073	0.002072	0.01592		0.02295	0.0191
Primary test-model 2 LTBI *v.* No LTBI (1 SNP)					0.04028		
SNPs associated with reduced TB risk (OR<1)	chr7:30498962(C) NOVEL VARIANT	chr7:30464249(TG) NOVEL VARIANT	rs5743370(C)	rs2288384			
Primary test-model 1 Active TB *v.* No Active TB (4 SNPs)	0.02847	0.01204	0.01204	0.002977			
Primary test-model 2 LTBI *v.* No LTBI (0 SNPs)

**Table 3.** Allele frequencies of TB phenotype-associated SNPs

Minor allele frequency distribution of phenotype-associated SNPs among sampled populations												
Gene	CHR	SNP	A1	A2	Combined		Merhabete		Adigrat		Arbaminch	
					F_A1-A	F_A1_U	F_A1-A	F_A1_U	F_A1-A	F_A1_U	F_A1-A	F_A1_U
*TICAM2*	5	chr5:114915999	A	C	0.026	0.004	0.016	0.000	0.013	0.000	0.036	0.009
	5	chr5:114916028	A	G	0.092	0.155	0.113	0.141	0.132	0.149	0.065	0.170
	5	chr5:114916090	G	A	0.464	0.417	0.371	0.436	0.447	0.457	0.506	0.368
*NOD1*	7	chr7:30464249	TG	T	0.007	0.018	0.000	0.000	0.000	0.000	0.012	0.047
	7	chr7:30464872	A	G	0.052	0.032	0.097	0.026	0.066	0.053	0.030	0.019
	7	chr7:30464932	G	T	0.010	0.018	0.016	0.000	0.000	0.043	0.012	0.009
	7	chr7:30465424	C	A	0.007	0.018	0.000	0.000	0.000	0.000	0.012	0.047
	7	chr7:30469270	C	T	0.219	0.198	0.113	0.128	0.250	0.149	0.244	0.293
	7	chr7:30477156	T	G	0.124	0.040	0.081	0.051	0.132	0.064	0.137	0.009
	7	chr7:30485722	T	G	0.121	0.032	0.048	0.026	0.118	0.053	0.149	0.019
	7	chr7:30490711	T	G	0.095	0.032	0.081	0.051	0.079	0.053	0.107	0.000
	7	chr7:30491081	A	G	0.043	0.011	0.017	0.000	0.013	0.011	0.066	0.019
	7	chr7:30498962	C	G	0.010	0.029	0.016	0.013	0.000	0.011	0.012	0.057

*Note*: A1/A2, minor/major allele; F_A1-A, frequency of minor allele in affecteds; F_A1-A, frequency of minor allele in unaffecteds.

### Adjustment for the effect of sex, age and population stratification (EGC)

While most SNPs survived covariate analyses (Supplementary Tables S6–S9), those SNPs with already moderate association signals in the logistic regression model failed to retain significance. This is most probably because of sample size reduction and a concomitant loss of power to detect associations. For example, the Merhabete-Adigrat EGCs together constitute the least sample size and Adigrat-Arbaminch the largest; similarly, there was a successive reduction in sample size in the test-model construction. However, one cannot ignore population stratification as a possible confounder.

### Accounting for possible population stratification

In as much as the actual ancestry of each individual can be inferred based on self-declared ethnicity or IBS, various tests were performed in order to directly assess and account for any possible stratification effect on the outcome of the association test statistic: population-specific tests of association (tests within each EGC); stratified analysis of association based on self-declared ethnicity (EGC) and based on assignment of individuals to homogeneous clusters based on IBS or empiric SNP data (Cochran–Mantel–Haenszel test); test for heterogeneous association (Breslow–Day test); and, test of allele frequency difference between EGCs (χ^2^ test) (Supplementary Tables S6–S10). Overall, while the top SNPs in this study associated with susceptibility phenotypes survived both the IBS and EGC-based tests of association, the IBS-based stratified analysis identified more SNP-phenotype association than the EGC-based test.

All phenotype-associated markers which survived the Bonferroni adjustment for multiple testing survived the FDR-adjustment as well (Supplementary Table S1^1^).

These results, coupled with the observation that the association of the top SNP in this study, rs751770147, was replicated in both the Adigrat and Arbaminch populations with relatively higher sample sizes but not in the Merhabete population with the least sample size, may indicate that the effect of sample size could be a more probable explanation for the varied test results of the population-specific association tests.

### LD structure

LD structure of the exonic regions was examined using Haploview which showed little LD in both genes suggesting a minor role for SNP co-segregation for the observed pattern of association (Figs 2 and 3 for NOD1 and TICAM2, respectively).

### Haplotype analyses

The omnibus haplotype association test, which examines the difference in haplotype distribution between cases and controls, was significant for TB *v.* no TB in *TICAM2* (*p* = 0.0178) and *NOD1* (*p* = 0.0381) (Supplementary Tables S12 and S13). This test statistic was particularly significant in the contrast between TB and LTBI for *NOD1* (*p* = 0.000359), providing additional evidence that this gene is associated with progression to active TB disease. In general, despite the apparently low LD between the TB phenotype-associated SNPs, the haplotype analysis further supports the single SNP association test results.

## Discussion

Our results replicated the association of *NOD1* and *TICAM2* genes with TB in Ethiopian populations. We found two variants in *NOD1* that were significantly associated with TB risk, and additional variants that were nominally associated. Furthermore, one of the most significantly associated SNPs in *NOD1* was novel. Though we did not observe a statistically significant association between TB and *TICAM2* variants after accounting for multiple testing, these nominally significant results still provide an independent replication of a previous report [23]. By contrasting TB disease with no disease and LTBI *v.* no LTBI, our study design helped to unravel a pattern of SNP-phenotype associations to determine which stage of TB pathogenesis was associated with these variants. In our analyses, we found consistent evidence of association of *NOD1* variants with active TB in both our primary and sensitivity test models, and two of these results were significant after multiple-test correction. We did not observe notable associations with LTBI as the case phenotype, except for a single nominally associated *NOD1* SNP, though some of the individuals in this category may progress to active TB later in time by virtue of possessing any of the susceptibility variants. The relatively smaller sample size in this test-model may also have reduced the power to detect association signals.

In addition to the careful phenotypic characterization of the control population, there are other aspects of this study that further enhance its validity. First, although other transcription-regulatory regions are also important, we focused on padded full exon resequencing of the two candidate genes of interest, enabling the discovery of novel genetic variants with a higher probability of translation. Second, we recruited individuals from multiple ethnic groups, and examined possible population stratification effects. It is hoped the design and conduct of this study, with particular emphasis on disease trait definitions that incorporate intermediate phenotypes, will serve as a genetic epidemiological model to future studies of complex disease traits. Although there were slight evidence suggestive of the presence of some population-specific genetic signatures, there was no strong indication of population stratification that would lead the authors of this study to put forward a recommendation of the prohibition of sampling from different ethno-geographic backgrounds and pooling data together particularly for studies based on exonic data of candidate genes.

Although it is typical in replication studies to perform a meta-analysis, we did not do so here for two reasons. First, to our best knowledge, this is only the first attempt at replication of these two genes. Second, we have taken an alternative approach that would make a meta-analysis with the data from the original work [23] nearly impossible. Rather than analyzing the same exact SNPs as in the original report, we conducted exon sequencing with some flanking buffers. Although this enabled us to identify novel variants in their association with TB, the gaps between the exons meant that we could not capture all the SNPs in the original study, i.e., the percentage of matching sequences between the two studies was low (6 SNPs in *TICAM2* and 34 SNPs in *NOD1*) which were further reduced by QC filtering. This effectively precluded a comprehensive comparative assessment at finer levels beyond the overall gene level. Nonetheless, we were able to capture one SNP in *NOD1*, rs17159043, which showed signals of association with TB in both the original study (unadjusted *p* value of 0.009) and the current replication study (unadjusted *p* value of 0.040 in LTBI *v.* No LTBI).

The relatively small sample size of this study is a limitation as it affects statistical inference and declaration of significance. Particularly, the progressively stricter definition of case-control phenotypes across test-model constructs comes at the cost of reduced sample size. This difference in sample size could explain in part the difference in the observed pattern of SNP-phenotype associations between the test-models. In addition, mycobacterial growth in culture, the gold standard for TB diagnosis, is not routinely used in Ethiopia, though the diagnostic criteria implemented are still quite rigorous. Lastly, there were quite a few subjects that tested HIV positive or had indeterminate Quantiferon results, which eliminated them from some analyses, thus reducing power. Therefore, future research with larger sample size is warranted.

In conclusion, we have provided an independent replication of association between TB and variants in *TICAM2* and *NOD1*. By conducting QFT to assess latent infection status, we were able to demonstrate that a higher proportion of these variants are associated with susceptibility to active TB disease, not a latent infection. Some variants in both genes were also associated with reduced risk to active TB. We examined multiple ethnic groups in Ethiopia, and found that our association results are robust to population stratification. We also identified novel variants in these genes. As Ethiopia is considered to be the origin of both humanity and *Mtb*, these findings are important for understanding *Mtb*-human co-evolution and the genetic underpinnings of TB in general. Our findings also support the hypothesis that the Ethiopian setting could indirectly help to explore the possibility that *Mtb*-human co-evolution may result in differential TB genetic risk profiles across population categories in that while our Ethiopian study replicated the Ugandan findings at the gene level, the associated SNPs in overlapping loci are not shared and some are novel. Therefore, while the Ugandan and Ethiopian findings indicate, on the one hand, a common signature of association at the gene level in the two populations, on the other hand, at the SNP level, the results suggest at the possible existence of population-specific signatures of association which may be driven by differential *Mtb*-human co-evolution. This phenomenon of population-specific adaptation is not uncommon and has been previously reported in Ethiopian populations with regard to genetic and physiological adaptations to high altitude (hypoxia) [54, 55]. The results of this study could encourage investigators who may be intimidated by the ‘burden’ of the high genetic diversity in African populations. The conduct of the study, interpretation of the results and any limitations should be informative to the efforts of the Human Heredity and Health in Africa initiative [56] at enriching region-specific health-related genomic knowledge.

## Supplementary material

For supplementary material accompanying this paper visit http://dx.doi.org/10.1017/gheg.2017.17. click here to view supplementary material

## References

[1] Ma N, Clinical and epidemiological charactersistics of individuals resistant to *M. tuberculosis* infection in a longitudinal TBHousehold contact study in Kampala, Uganda. BMC Infectious Diseases 2014; 14: 352. 24970328 10.1186/1471-2334-14-352 PMC4091673

[2] World-Health-Organization. *Global Tuberculosis Report, 2015* 2015 www.who/int/tb/data

[3] Smith I. *Mycobacterium tuberculosis* pathogenesis and molecular determinants of virulence. Clinical Microbiology Reviews 2003; 16: 463–496. 12857778 10.1128/CMR.16.3.463-496.2003 PMC164219

[4] Van Crevel R, Ottenhoff THM, Van der Meer JWM. Innate immunity to *Mycobacterium tuberculosis*. Clinical Microbiology Reviews 2002; 15: 294–309. 11932234 10.1128/CMR.15.2.294-309.2002 PMC118070

[5] Ernst JD, Trevejo-Nunez G, Banaiee N. Genomics and the evolution, pathogenesis, and diagnosis of tuberculosis. The Journal of Clinical Investigation 2007; 117: 1738–1745. 17607348 10.1172/JCI31810 PMC1904327

[6] Filliol I, Global phylogeny of *Mycobacterium tuberculosis* based on single nucleotide polymorphism (SNP) analysis: insights into tuberculosis evolution, phylogenetic accuracy of other DNA fingerprinting systems, and recommendations for a minimal standard SNP set. Journal of Bacteriology 2006; 188: 759–772. 16385065 10.1128/JB.188.2.759-772.2006 PMC1347298

[7] Stein CM. Genetic epidemiology of tuberculosis susceptibility: impact of study design. PLoS Pathogens 2011; 7: e1001189. 21283783 10.1371/journal.ppat.1001189 PMC3024264

[8] Stein CM, Linkage and association analysis of candidate genes for TB and TNFalpha cytokine expression: evidence for association with IFNGR 1, IL-10, and TNF receptor 1 genes. Human Genetics 2007; 121: 663–673. 17431682 10.1007/s00439-007-0357-8

[9] Glassroth J. Tuberculosis 2004: challenges and opportunities. Transactions of the American Clinical and Climatological Association 2005; 116: 293–310. 16555622 PMC1473147

[10] Azad AK, Sadee W, Schlesinger LS. Innate immune gene polymorphisms in tuberculosis. Infection and Immunity 2012; 80: 10. 10.1128/IAI.00443-12 PMC3457569 22825450

[11] Sirugo G, Genetic studies of African populations: an overview on disease susceptibility and response to vaccines and therapeutics. Human Genetics 2008; 123: 557–598. 18512079 10.1007/s00439-008-0511-y

[12] Galagan JE. Genomic insights into tuberculosis. Nature Rviews/Genetics 2014; 15: 307–317. 10.1038/nrg3664 24662221

[13] Marlo M, Eileen GH. Current findings, challenges and novel approaches in human genetic susceptibility to tuberculosis. Tuberculosis 2010; 90: 71–83. 20206579 10.1016/j.tube.2010.02.002

[14] Hill AV. Aspects of genetic susceptibility to human infectious diseases. Annual Review of Genetics 2006; 40: 469–486. 10.1146/annurev.genet.40.110405.090546 17094741

[15] Awomoyi AA, Interleukin-10, polymorphism in SLC11A1 (formerly NRAMP1), and susceptibility to tuberculosis. Journal of Infectious Diseases 2002; 186: 1808–1814. 12447767 10.1086/345920

[16] Bellamy R, Variations in the NRAMP1 gene and susceptibility to tuberculosis in West Africans. New England Journal of Medicine 1998; 338: 640–644. 9486992 10.1056/NEJM199803053381002

[17] Awomoyi AA, Polymorphism in IL1B: IL1B-511 association with tuberculosis and decreased lipopolysaccharide-induced IL-1beta in IFN-gamma primed ex-vivo whole blood assay. Journal of Endotoxin Research 2005; 11: 281–286. 16263000 10.1179/096805105X58706

[18] Bornman L, Vitamin D receptor polymorphisms and susceptibility to tuberculosis in West Africa: a case-control and family study. Journal of Infectious Diseases 2004; 190: 1631–1641. 15478069 10.1086/424462

[19] Lombard Z, Association of HLA-DR, -DQ, and vitamin D receptor alleles and haplotypes with tuberculosis in the Venda of South Africa. Human Immunology 2006; 67: 643–654. 16916662 10.1016/j.humimm.2006.04.008

[20] Olesen R, DC-SIGN (CD209), pentraxin 3 and vitamin D receptor gene variants associate with pulmonary tuberculosis risk in West Africans. Genes and Immunity 2007; 8: 456–467. 17611589 10.1038/sj.gene.6364410

[21] Li CM, Association of a polymorphism in the P2X7 gene with tuberculosis in a Gambian population. Journal of Infectious Diseases 2002; 186: 1458–1462. 12404161 10.1086/344351

[22] Bellamy R, Genetic susceptibility to tuberculosis in Africans: a genome-wide scan. Proceedings of the National Academy of Sciences of the United States of America 2000; 97: 8005–8009. 10859364 10.1073/pnas.140201897 PMC16660

[23] Hall NB, Polymorphisms in TICAM2 and IL1B are associated with TB. Genes and Immunity 2015; 16: 127–133. 25521228 10.1038/gene.2014.77 PMC4352113

[24] Malik S, Variants of the SFTPA1 and SFTPA2 genes and susceptibility to tuberculosis in Ethiopia. Human Genetics 2005; 118: 752–759. 16292672 10.1007/s00439-005-0092-y

[25] Hiroyuki T, Synergistic effect of Nod1 and Nod2 agonists with Toll-Like receptor agonists on human dendritic cells to generate interleukin-12 and T helper type 1 cells. Infection and Immunity 2005; 73: 12. 10.1128/IAI.73.12.7967-7976.2005 PMC1307098 16299289

[26] Elson G. Contribution of Toll-like receptors to innate immune responses to gram-negative and gram-positive bacteria. The American Journal of Hematology 2007; 109: 4. 10.1182/blood-2006-06-032961 17038528

[27] Takeda K, Akir S. Toll-like receptors in innate immunity. The Japaneees Society for Immunology 2005; 17: 1. 10.1093/intimm/dxh186 15585605

[28] Seya T, TICAM-1 and TICAM-2: toll-like receptor adapters that participate in induction of type 1 interferons. International Journal of Biochemistry & Cell Biology 2005; 37: 3. 10.1016/j.biocel.2004.07.018 15618008

[29] Yoshiaki E, Structures and interface mapping of the TIR domaincontaining adaptor molecules involved ininterferon signaling. Proceedings of the National Academy of Sciences 2013; 110: 49. 10.1073/pnas.1222811110 PMC3856793 24255114

[30] Magali M, Roles for Treg Expansion and HMGB1 Signaling through the TLR1-2-6 axis in determining the magnitude of the antigen-specific immune response to MVA85A. PLOS ONE 2013; 8: 7. 10.1371/journal.pone.0067922 PMC3700883 23844129

[31] Lilian OM, Dario SZ. NOD1 and NOD2 signaling in infection and inflamation. Frontiers in Immunology 2012; 3: 28. 23162548 10.3389/fimmu.2012.00328 PMC3492658

[32] Luigi F, Intracellular NOD-like receptors in innate immunity, infection and disease. Cellular Microbiology 2008; 10: 1. 17944960 10.1111/j.1462-5822.2007.01059.x

[33] Esmeralda J, Nucleotide-oligomerizing domain-1 (NOD1) receptor activation induces pro-inflammatory responses and autophagy in human alveolar macrophages. BMC Pulmonary Medicine 2014; 4: 152. 10.1186/1471-2466-14-152 PMC4190423 25253572

[34] Jun-young L, The role of nucleotide-binding oligomerization domain 1 (NOD1) in cytokine production by macrophages in response to *Mycobacterium tuberculosis*. In Conference: *2*nd Annual Meeting of the International-Cytokine-and-Interferon – Society, 2014, vol. 70.

[35] Pagani L, Ethiopian genetic diversity reveals linguistic stratification and complex influences on the Ethiopian gene pool. The American Journal of Human Genetics 2012; 91: 83–96. 22726845 10.1016/j.ajhg.2012.05.015 PMC3397267

[36] Pagani L, Tracing the route of modern humans out of Africa by using 225 human genome sequences from Ethiopians and Egyptians. The American Journal of Human Genetics 2015; 96: 1–5. 10.1016/j.ajhg.2015.04.019 PMC4457944 26027499

[37] Semino O, Ethiopians and Khoisan share the deepest clades of the human Y-chromosome phylogeny. American Journal of Human Genetics 2002; 70: 265–268. 11719903 10.1086/338306 PMC384897

[38] Kivisild K, Ethiopian mitochondrial DNA heritage: tracking gene flow across and around the gate of tears. American Journal of Human Genetics 2004; 75: 752–770. 15457403 10.1086/425161 PMC1182106

[39] Lovell A, Ethiopia: between Sub-Saharan Africa and western Eurasia. Annals of Human Genetics 2005; 69: 275–287. 15845032 10.1046/J.1469-1809.2005.00152.x

[40] Liu H, A geographically explicit genetic model of worldwide human-settlement history. The American Journal of Human Genetics 2006; 79: 230–237. 16826514 10.1086/505436 PMC1559480

[41] Poloni ES, Genetic evidence for complexity in ethnic differentiation and history in East Africa. Annals of Human Genetics 2009; 73; 582–600. 19706029 10.1111/j.1469-1809.2009.00541.x

[42] Gurdasani D, The African genome variation project shapes medical genetics in Africa. Nature 2014; 517: 327–332. 25470054 10.1038/nature13997 PMC4297536

[43] Gagneux S. Host-pathogen coevolution in human tuberculosis. Philosophical Transactions of the Royal Society of London B: Biological Sciences 2012; 367: 850–859. 22312052 10.1098/rstb.2011.0316 PMC3267123

[44] Vermund S, Yamamoto N. Co-infection with human immunodeficiency virus and tuberculosis in Asia. Tuberculosis *(*Edinb*)*, 2007; 87: 18–25. 10.1016/j.tube.2007.05.008 PMC2031213 17631414

[45] Nahid P, Treatment outcomes of patients with HIV and tuberculosis. American Journal of Respiratory and Critical Care Medicine 2007; 175: 11. 10.1164/rccm.200509-1529OC PMC1899273 17290042

[46] Rice JP, Definition of the Phenotype. Advances in Genetics. Academic Press; 2001, vol. 42. 10.1016/s0065-2660(01)42015-3 11037314

[47] Rebuma F, Mycobacterial lineages causing pulmonary and extrapulmonary tuberculosis, Ethiopia. Emerging Infectious Diseases 2013; 19: 3. 10.3201/eid1903.120256 PMC3647644 23622814

[48] Yimer SA, Mycobacterium tuberculosis lineage 7 strains are associated with prolonged patient delay in seeking treatment for pulmonary tuberculosis in Amhara Region, Ethiopia. Journal of Clinical Microbiology, 2015; 53: 4. 25673798 10.1128/JCM.03566-14 PMC4365194

[49] Mulugeta B, Strain diversity of *Mycobacterium tuberculosis* isolates from pulmonary tuberculosis patients in afar pastoral region of Ethiopia. BioMed Research International 2014; 2014. doi: 10.1155/2014/238532. PMC3966356 24734230

[50] Federal Ministry of Health of Health, Ethiopia. Tuberculosis, Leprosy and TB/HIV Prevention and Control Programme *(*Manual*)*. 2005.

[51] Federal Ministry of Health of Health, Ethiopia. Manual of the National Tuberculosis and Leprosy Control Program, Ethiopia. 1997.

[52] Mengistu MM, Tesfaye WT, Madeley JR. The quality of tuberculosis diagnosis in districts of Tigray region of northern Ethiopia. The Ethiopian Journal of Health Development 2005; 19: 13–20.

[53] Purcell S, PLINK (1.07) Documentation: a toolset for whole-genome association and population-based linkage analysis. URL: http://pngu.mgh.harvard.edu/purcell/plink/ [Online], 2007. 10.1086/519795 PMC1950838 17701901

[54] Beall CM, An Ethiopian pattern of human adaptation to high-altitude hypoxia. Proceedings of the National Academy of Sciences 2002; 99: 17215–17218. 10.1073/pnas.252649199 PMC139295 12471159

[55] Scheinfeldt LB, Genetic adaptation to high altitude in the Ethiopian highlands. Genome Biology 2012; 13: R1(http://genomebiology.com/2012/13/1/R1) 10.1186/gb-2012-13-1-r1 PMC3334582 22264333

[56] H3Africa Consortium. Research capacity: enabling the genomic revolution in Africa. Science 2014; 344: 1346–1348. 24948725 10.1126/science.1251546 PMC4138491

