# Supplementary material for: Novel polymorphisms in TICAM2 and NOD1 associated with tuberculosis progression phenotypes in Ethiopian populations
Source: Glob Health Epidemiol Genom. 2018 Jan 23;3:e1. doi: 10.1017/gheg.2017.17 (PMC5870410; doi:10.1017/gheg.2017.17)
Supplement: Supplementary file 1 [file S2054420017000173sup001.docx]

Supplementary Table S1. Results of Interferon-Gamma Release Assay (IGRA)

| Summary of latent TB infection (LTBI) test result | | | | | | | |
| --- | --- | --- | --- | --- | --- | --- | --- |
| Sampling site | Indeterminate | % | Negative | % | Positive | % | Total |
| Adigrat | 0 | 0 | 26 | 48 | 28 | 52 | 54 |
| Arbaminch | 7 | 11 | 21 | 32 | 38 | 58 | 66 |
| Merhabete | 1 | 2 | 28 | 58 | 19 | 40 | 48 |
| Total | 8 | 5 | 75 | 45 | 85 | 51 | 168 |

Supplementary Table S2: Description of genome coordinates for candidate gene regions

| Description of genome coordinates for candidate gene regions (exons with padding) captured for sequencing using Homosapiens/UCSC/hg19 | | | | | | | | | |
| --- | --- | --- | --- | --- | --- | --- | --- | --- | --- |
| Target | Chromosome | Start Coordinate | Stop Coordinate | Length | Padding Per Exon | Amplicons | SNP Count | Coverage | Score |
| TICAM2_Exon_2233011 | 5 | 114937769 | 114938201 | 433 | 25 | 2 | 2 | 100 | 70 |
| TICAM2_Exon_2236359 | 5 | 114914314 | 114917037 | 2724 | 25 | 10 | 1136 | 100 | 74 |
| NOD1_Exon_2252938 | 7 | 30475589 | 30475722 | 134 | 25 | 1 | 0 | 100 | 96 |
| NOD1_Exon_2253679 | 7 | 30518197 | 30518418 | 222 | 25 | 1 | 2 | 100 | 60 |
| NOD1_Exon_2254637 | 7 | 30486558 | 30486691 | 134 | 25 | 1 | 0 | 100 | 96 |
| NOD1_Exon_2253753 | 7 | 30490807 | 30492681 | 1875 | 25 | 7 | 14 | 100 | 65 |
| NOD1_Exon_2253177 | 7 | 30472687 | 30472820 | 134 | 25 | 1 | 4 | 100 | 96 |
| NOD1_Exon_2254763 | 7 | 30499492 | 30499682 | 191 | 25 | 1 | 4 | 100 | 96 |
| NOD1_Exon_2254950 | 7 | 30487889 | 30488022 | 134 | 25 | 1 | 0 | 100 | 80 |
| NOD1_Exon_2253131 | 7 | 30485732 | 30485865 | 134 | 25 | 1 | 0 | 100 | 96 |
| NOD1_Exon_2253178 | 7 | 30494728 | 30494952 | 225 | 25 | 1 | 0 | 100 | 96 |
| NOD1_Exon_2253752 | 7 | 30477164 | 30477297 | 134 | 25 | 1 | 0 | 100 | 80 |
| NOD1_Exon_2254373 | 7 | 30468965 | 30469098 | 134 | 25 | 1 | 0 | 100 | 96 |
| NOD1_Exon_2254638 | 7 | 30498743 | 30498881 | 139 | 25 | 1 | 2 | 100 | 96 |
| NOD1_Exon_2254949 | 7 | 30464118 | 30465351 | 1234 | 25 | 5 | 4 | 100 | 83 |
| NOD1_Exon_2254951 | 7 | 30496312 | 30496683 | 372 | 25 | 2 | 6 | 100 | 80 |

**Supplementary Table S3: Summary of exonic sequence data before and after quality control filtering**

| Quality Control (QC) | | | | | | | | | | | | | |
| --- | --- | --- | --- | --- | --- | --- | --- | --- | --- | --- | --- | --- | --- |
|  |  | Before QC Filtering | | | | | | | | After QC Filtering | | | |
| Test-model | n | *A high proportion of the markers showed no variation among the samples (Monomorphic Markers=MM)* | | | | | | | | *-Per-individual QC: <90% Genotyping Rate (GR) -Per-marker QC:<95% GR; Minor Allele Frequency (MAF):<0.01; Hardy-Weinberg-Equilibrium (HWE): p<0.001 (Tests for significant bias in genotyping rate between cases and controls were non-significant)* | | | |
|  |  | TICAM2 | | | | NOD1 | | | | TICAM2 | | NOD1 | |
|  |  | #Markers | GR | MM | %MM | #Markers | GR | MM | %MM | #Markers | GR | #Markers | GR |
| Active TB  vs.  No Active TB | 292 | 3578 | 0.86 | 3343 | 93 | 8349 | 0.92 | 7386 | 88 | 12 | 1 | 24 | 0.99 |
| Active TB  vs.  No LTBI | 217 | 3578 | 0.86 | 3406 | 95 | 8348 | 0.92 | 7619 | 91 | 12 | 1 | 21 | 0.99 |
| Active TB  vs.  LTBI | 223 | 3577 | 0.86 | 3374 | 94 | 8348 | 0.92 | 7576 | 91 | 12 | 1 | 24 | 0.99 |
| LTBI  vs.  No LTBI | 134 | 3575 | 0.86 | 3432 | 96 | 8343 | 0.92 | 7830 | 94 | 13 | 1 | 23 | 0.99 |
| Average |  | 3577 | 0.86 | 3389 | 95 | 8347 | 0.92 | 7603 | 91 | 12 | 1 | 23 | 0.99 |

Supplementary Table S4: NOD1 and TICAM2 SNPs associated with increased risk to TB progression phenotypes

| Best results in Active TB vs. No Active TB: SNPs Associated With Susceptibility to Active TB (Primary test-model 1) | | | | | | | |
| --- | --- | --- | --- | --- | --- | --- | --- |
| Gene | SNP | A1 | P | BONF. | OR | (L95 | U95) |
| *NOD1* | chr7:30485722 | T | 7.28E-05 | 0.006841 | 4.111 | 1.946 | 8.684 |
|  | chr7:30477156 | T | 0.000104 | 0.009539 | 16.66 | 2.214 | 125.3 |
|  | chr7:30490711 | T | 0.002072 |  | 3.457 | 1.545 | 7.733 |
|  | chr7:30491081 | A | 0.01592 |  | 4.519 | 1.218 | 16.77 |
| *TICAM2* | chr5:114915999 | A | 0.0191 |  | 9.995 | 1.071 | 93.26 |
|  | chr5:114916090 | G | 0.02295 |  | 1.785 | 1.079 | 2.955 |
| Best results in Active TB vs. No LTBI: SNPs Associated With Susceptibility to Active TB (Sensitivity analysis) | | | | | | | |
| Gene | SNP | A1 | P | BONF. | OR | (L95 | U95) |
| *NOD1* | chr7:30485722 | T | 0.0001597 | 0.0131 | 8.665 | 2.056 | 36.52 |
|  | chr7:30477156 | T | 0.008836 |  | 3.445 | 1.365 | 8.696 |
|  | chr7:30490711 | T | 0.01889 |  | 3.717 | 1.242 | 11.12 |
| Best results in Active TB vs. LTBI: SNPs Associated With Susceptibility to Active TB (Sensitivity analysis) | | | | | | | |
| Gene | SNP | A1 | P | BONF. | OR | (L95 | U95) |
| *NOD1* | chr7:30477156 | T | 0.002353 |  | 9.804 | 2.252 | 42.68 |
|  | chr7:30485722 | T | 0.004529 |  | 4.892 | 1.635 | 14.64 |
|  | chr7:30490711 | T | 0.01477 |  | 6.33 | 1.436 | 27.9 |
|  | chr7:30491081 | A | 0.04496 |  | 6.253 | 0.8098 | 48.28 |
|  | chr7:30464872 | A | 0.04505 |  | 4.012 | 0.9193 | 17.51 |
| *TICAM2* | chr5:114916090 | G | 0.04545 |  | 1.826 | 1.008 | 3.305 |
| Best results in LTBI vs. No LTBI: SNPs Associated With Susceptibility to LTBI (Primary test-model 2) | | | | | | | |
| Gene | SNP | A1 | P | BONF. | OR | (L95 | U95) |
| *NOD1* | chr7:30469270 | C | 0.04028 |  | 1.947 | 1.03 | 3.681 |

BONF.=Bonferroni-corrected p-value; OR=Odds Ratio; L95/U95= Lower/Upper boundary of 95% Confidence Interval

Supplementary Table S5: NOD1 and TICAM2 SNPs associated with reduced risk to TB progression phenotypes

| Best results in Active TB vs. No Active TB: SNPs Associated With Resistance to Active TB (Primary test-model 1) | | | | | | | |
| --- | --- | --- | --- | --- | --- | --- | --- |
| Gene | SNP | A1 | P | BONF. | OR | (L95 | U95) |
| *NOD1* | chr7:30464249 | TG | 0.01204 |  | 0.1031 | 0.01448 | 0.7347 |
|  | chr7:30465424 | C | 0.01204 |  | 0.1031 | 0.01448 | 0.7347 |
|  | chr7:30498962 | C | 0.02847 |  | 0.162 | 0.02809 | 0.9338 |
| *TICAM2* | chr5:114916028 | A | 0.002977 |  | 0.3046 | 0.1346 | 0.6894 |
| Best results in Active TB vs. No LTBI: SNPs Associated With Resistance to Active TB (Sensitivity analysis) | | | | | | | |
| Gene | SNP | A1 | P | BONF. | OR | (L95 | U95) |
| *NOD1* | chr7:30498962 | C | 0.01872 |  | 0.2081 | 0.04735 | 0.9148 |
|  | chr7:30464932 | G | 0.02456 |  | 0.14 | 0.02521 | 0.7771 |
| *TICAM2* | chr5:114916028 | A | 0.003078 |  | 0.4183 | 0.2306 | 0.7588 |
| Best results in Active TB vs. LTBI: SNPs Associated With Resistance to Active TB (Sensitivity analysis) | | | | | | | |
| Gene | SNP | A1 | P | BONF. | OR | (L95 | U95) |
| No significant associations observed | | | | | | | |
| Best results in LTBI vs. No LTBI: SNPs Associated With Resistance to LTBI (Primary test-model 2) | | | | | | | |
| Gene | SNP | A1 | P | BONF. | OR | (L95 | U95) |
| No significant associations observed | | | | | | | |

BONF.=Bonferroni-corrected p-value; OR=Odds Ratio; L95/U95= Lower/Upper boundary of 95% Confidence Interval

Supplementary Table S6. Association test results in Test-model 1

| Tests of SNP-phenotype association: "Active TB" vs. "No Active TB" | | | | | | | | | | | | | | | | | | | | | | | | | | |
| --- | --- | --- | --- | --- | --- | --- | --- | --- | --- | --- | --- | --- | --- | --- | --- | --- | --- | --- | --- | --- | --- | --- | --- | --- | --- | --- |
| Gene | SNP | Minor allele | Best p | OR | Fisher | | | | Pearson | | | | Logistic regression | | | | Covariate analyses | | | | | Stratified tests (CMH) | | | | |
|  |  |  |  |  | Combined | Merhabete | Adigrat | Arbaminch | Combined | Merhabete | Adigrat | Arbaminch | Combined | Merhabete | Adigrat | Arbaminch | Sex | Age | Mer-Adi | Mer-Arb | Adi-Arb | EGC | IBS | IBS-Mer | IBS-Adi | IBS-Arb |
| *TICAM2* | chr5:114915999 | A | 1.91E-02 | 10.0 | 3.96E-02 |  |  |  | 2.72E-02 |  |  |  |  |  |  |  |  |  |  |  |  |  | 1.91E-02 |  |  |  |
|  | chr5:114916090 | G | 2.30E-02 | 1.8 |  |  |  | 3.39E-02 |  |  |  | 2.54E-02 |  |  |  | 2.95E-02 |  |  |  |  |  |  |  |  |  | 2.30E-02 |
|  | *chr5:114916028* | *A* | *2.98E-03* | *0.30* | *2.24E-02* |  |  | *8.38E-03* | *1.96E-02* |  |  | *6.25E-03* | *2.52E-02* |  |  | *1.35E-02* | *2.82E-02* | *2.72E-02* |  | *2.04E-02* | *3.40E-02* | *2.96E-02* | *1.55E-02* |  |  | *2.98E-03* |
| *NOD1* | chr7:30477156 | T | 1.04E-04* | 16.7 | 2.61E-04 |  |  | 1.04E-04 | 2.30E-04 |  |  | 2.78E-04 | 2.31E-04 |  |  | 4.17E-03 | 2.54E-04 | 1.73E-04 |  | 8.82E-04 | 2.21E-04 | 2.36E-04 | 4.85E-04 |  |  | 6.07E-04 |
|  | chr7:30485722 | T | 7.28E-05* | 4.1 | 8.25E-05 |  |  | 2.58E-04 | 7.28E-05 |  |  | 4.40E-04 | 1.02E-04 |  |  | 1.72E-03 | 8.69E-05 | 7.57E-05 |  | 8.90E-04 | 2.32E-04 | 1.76E-04 | 9.69E-05 |  |  | 1.44E-03 |
|  | chr7:30490711 | T | 2.07E-03 | 3.5 | 2.29E-03 |  |  |  | 2.26E-03 |  |  |  | 2.44E-03 |  |  |  | 2.65E-03 | 2.08E-03 |  | 2.14E-03 | 2.38E-03 | 2.07E-03 | 6.63E-03 |  |  |  |
|  | chr7:30491081 | A | 1.59E-02 | 4.5 | 2.12E-02 |  |  |  | 1.78E-02 |  |  |  | 3.73E-02 |  |  |  | 4.81E-02 | 4.50E-02 |  |  |  |  | 1.59E-02 |  |  |  |
|  | *chr7:30464249* | *TG* | *1.20E-02* | *0.10* |  |  |  |  |  |  |  |  |  |  |  |  |  |  |  |  |  |  |  |  |  | *1.20E-02* |
|  | *chr7:30465424* | *C* | *1.20E-02* | *0.10* |  |  |  |  |  |  |  |  |  |  |  |  |  |  |  |  |  |  |  |  |  | *1.20E-02* |
|  | *chr7:30498962* | *C* | *2.85E-02* | *0.16* |  |  |  |  |  |  |  | *3.23E-02* |  |  |  | *4.79E-02* |  |  |  |  | *3.39E-02* | *4.41E-02* |  |  |  | *2.85E-02* |

CMH=Cochran-Mantel-Haenszel test; EGC= Ethno-Geographic-Categories (Merhabete, Adigrat, and Arbaminch); Mer-Adi=Merhabete-Adigrat; Mer-Arb=Merhabete-Arbaminch; Adigrat-Arbaminch pair-wise (population covariates); IBS=Identity-by-Descent

**Supplementary Table S7**. Association test results in Test-model 2

| Tests of SNP-phenotype association: "Active TB" vs. "No LTBI" | | | | | | | | | | | | | | | | | | | | | | | | | | |
| --- | --- | --- | --- | --- | --- | --- | --- | --- | --- | --- | --- | --- | --- | --- | --- | --- | --- | --- | --- | --- | --- | --- | --- | --- | --- | --- |
| Gene | SNP | Minor allele | Best p | OR | Fisher | | | | Pearson | | | | Logistic regressions | | | | Covariate analysis | | | | | Stratified tests (CMH) | | | | |
|  |  |  |  |  | Combined | Merhabete | Adigrat | Arbaminch | Combined | Merhabete | Adigrat | Arbaminch | Combined | Merhabete | Adigrat | Arbaminch | Sex | Age | Mer-Adi | Mer-Arb | Adi-Arb | EGC | IBS | IBS-Mer | IBS-Adi | IBS-Arb |
| *TICAM2* | *chr5:114916028* | *A* | *3.08E-03* | *0.42* | *8.79E-03* |  |  | *1.39E-02* | *4.98E-03* |  |  | *7.95E-03* | *6.24E-03* |  |  | *1.43E-02* | *6.91E-03* | *6.86E-03* |  | *2.06E-02* | *1.64E-02* | *1.43E-02* | *3.08E-03* |  |  |  |
| *NOD1* | chr7:30477156 | T | 8.84E-03 | *3.4* | 1.43E-02 |  |  |  | 1.50E-02 |  |  | 4.65E-02 | 1.31E-02 |  |  |  | 1.33E-02 | 8.84E-03 |  | 2.48E-02 | 4.12E-02 | 2.21E-02 | 1.82E-02 |  |  |  |
|  | chr7:30485722 | T | 1.60E-04* | *8.7* | 1.60E-04 |  |  | 3.31E-02 | 4.70E-04 |  |  | 3.34E-02 | 2.04E-03 |  |  | 4.77E-02 | 2.03E-03 | 1.34E-03 | 4.08E-02 | 2.54E-02 | 7.91E-03 | 1.65E-03 | 3.84E-04 |  | 4.74E-02 |  |
|  | chr7:30490711 | T | 1.89E-02 | *3.7* | 2.73E-02 |  |  |  | 2.28E-02 |  |  |  | 2.40E-02 |  |  |  | 2.54E-02 | 1.89E-02 |  | 3.07E-02 |  | 3.00E-02 | 2.90E-02 |  |  |  |
|  | *chr7:30464932* | *G* | *2.46E-02* | *0.14* |  |  |  |  | *3.88E-02* |  |  | *1.92E-02* |  |  |  |  |  | *4.92E-02* |  |  | *2.46E-02* | *4.84E-02* |  |  |  |  |
|  | *chr7:30498962* | *C* | *1.87E-02* | *0.21* |  |  |  |  | *3.88E-02* |  |  |  |  |  |  | *3.74E-02* |  | *4.85E-02* |  |  | *1.89E-02* | *1.87E-02* | *3.38E-02* |  |  |  |

**Supplementary Table S8**. Association test results in Test-model 3

| Tests of SNP-phenotype association: "Active TB" vs. "LTBI" | | | | | | | | | | | | | | | | | | | | | | | | | | |  |
| --- | --- | --- | --- | --- | --- | --- | --- | --- | --- | --- | --- | --- | --- | --- | --- | --- | --- | --- | --- | --- | --- | --- | --- | --- | --- | --- | --- |
| Gene | SNP | Minor allele | Best p | OR | Fisher | | | | Pearson | | | | Logistic regression | | | | Covariate analysis | | | | | Stratified tests (CMH) | | | | | |
|  |  |  |  |  | Combined | Merhabete | Adigrat | Arbaminch | Combined | Merhabete | Adigrat | Arbaminch | Combined | Merhabete | Adigrat | Arbaminch | Sex | Age | Mer-Adi | Mer-Arb | Adi-Arb | EGC | IBS | IBS-Mer | IBS-Adi | IBS-Arb | |
| *TICAM2* | chr5:114916090 | G | 4.55E-02 | 1.8 |  |  |  |  |  |  |  | 4.55E-02 |  |  |  |  |  |  |  |  |  |  |  |  |  |  | |
| *NOD1* | chr7:30464872 | A | 4.51E-02 | 4.0 |  |  |  |  |  |  |  |  |  |  |  |  |  |  |  |  |  |  | 4.51E-02 |  |  |  | |
|  | chr7:30477156 | T | 2.35E-03 | 9.8 | 2.88E-03 |  |  |  | 3.31E-03 |  |  |  | 3.58E-03 |  |  | 2.35E-03 | 3.95E-03 | 3.65E-03 |  | 1.36E-02 |  | 3.38E-03 | 3.34E-03 |  |  |  | |
|  | chr7:30485722 | T | 4.53E-03 | 4.9 | 9.07E-03 |  |  |  | 9.54E-03 |  |  |  | 8.72E-03 |  |  |  | 7.59E-03 | 8.91E-03 |  | 8.92E-03 | 4.53E-03 | 1.04E-02 | 1.23E-02 |  |  |  | |
|  | chr7:30490711 | T | 1.48E-02 | 6.3 | 3.35E-02 |  |  |  | 2.92E-02 |  |  |  | 2.86E-02 |  |  |  | 3.01E-02 | 2.92E-02 |  | 3.75E-02 | 1.48E-02 | 3.04E-02 | 4.09E-02 |  |  |  | |
|  | chr7:30491081 | A | 4.50E-02 | 6.3 |  |  |  |  | 4.50E-02 |  |  |  |  |  |  |  |  |  |  |  |  |  | 4.97E-02 |  |  |  | |

Supplementary Table S9. Association test results in Test-model 4

|  | | Tests of SNP-phenotype association: "LTBI" vs. "No LTBI" | | | | | | | | | | | | | | | | | | | | | | | | | |
| --- | --- | --- | --- | --- | --- | --- | --- | --- | --- | --- | --- | --- | --- | --- | --- | --- | --- | --- | --- | --- | --- | --- | --- | --- | --- | --- | --- |
| Gene | SNP | | Minor allele | Best p | OR | Fisher | | | | Pearson | | | | Logistic regression | | | | Covariate analysis | | | | | Stratified tests (CMH) | | | | |
|  |  |  |  |  |  | Combined | Merhabete | Adigrat | Arbaminch | Combined | Merhabete | Adigrat | Arbaminch | Combined | Merhabete | Adigrat | Arbaminch | Sex | Age | Mer-Adi | Mer-Arb | Adi-Arb | EGC | IBS | IBS-Mer | IBS-Adi | IBS-Arb |
| *NOD1* | chr7:30469270 | | C | 4.03E-02 | 1.9 |  |  |  |  |  |  |  |  |  |  |  |  | 4.03E-02 |  |  |  |  |  | 4.08E-02 |  |  |  |

Supplementary Table S10. Test for heterogeneous association between populations

| Heterogeneous association between ethno-geographic categories | | | | | | | | | |
| --- | --- | --- | --- | --- | --- | --- | --- | --- | --- |
| Gene | Test-model | SNP | A1 | MAF | CHISQ | P | OR | CHISQ_BD | P_BD |
| *NOD1* | Active TB vs. No Active TB | chr7:30490711 | T | 0.06507 | 9.484 | 0.002072 | 3.457 | 6.674 | 0.03555 |
|  | Active TB vs. LTBI | chr7:30477156 | T | 0.09641 | 8.591 | 0.003378 | 3.901 | 7.344 | 0.02542 |
|  |  | chr7:30485722 | T | 0.09641 | 6.564 | 0.0104 | 3.111 | 7.999 | 0.01832 |

Supplementary Table S11: Comparison of p-values before and after adjustment for multiple testing for all phenotype-associated SNPs

p-values were adjusted by three methods implemented in PLINK: Bonferroni method (Bonf,-adjusted); Benjamini & Hochberg method; and, Benjamini & Yekutieli method(FDR-BY-adjusted)

**Supplementary Table S12**. Results of haplotype-based association analysis for the *TICAM2* gene

| **Conditional haplotype test results : TICAM2 Gene (only significant results are shown)** | | | |
| --- | --- | --- | --- |
| **HAPLOTYPES: Active TB vs. No Active TB** | **FREQ** | **P: Haplotype-specific test** | **P: After controlling specific haplotypes** |
| **AGG** | **0.0154** | **0.017** | **0.112** |
| **CAA** | **0.122** | **0.0225** | **0.0871** |
| **CGG** | **0.426** | **0.572** | **0.00757** |
| **CGA** | **0.437** | **0.708** | **0.00692** |
| **Likelihood ratio test: chi-square =10.1** |  |  |  |
| **df = 3** |  |  |  |
| **Omnibus p = 0.0178** |  |  |  |

FREQ=Haplotype frequency

Supplementary Table S13. Results of haplotype-based association analysis in the *NOD1* gene

| **Conditional haplotype test results : NOD1 Gene (only significant results are shown)** | | | |
| --- | --- | --- | --- |
| **HAPLOTYPES: Active TB vs. No Active TB** | **FREQ** | **P: Haplotype-specific test** | **P: After controlling specific haplotypes** |
| **TGTATGGGGG** | **0.63** | **0.000711** | **0.867** |
| **TGTATTTGGG** | **0.0209** | **0.0138** | **0.201** |
| **TGTATGGGAG** | **0.0142** | **0.039** | **0.106** |
| **Likelihood ratio test: chi-square = 13.3** |  |  |  |
| **df = 6** |  |  |  |
| **Omnibus p = 0.0381** |  |  |  |
| **HAPLOTYPES: Active TB vs. No LTBI** | **FREQ** | **P: Haplotype-specific test** | **P: After controlling specific haplotypes** |
| **GTTGGGGG** | **0.614** | **0.0065** | **0.309** |
| **GTCTTTGG** | **0.0163** | **0.0168** | **0.19** |
| **GTTTGGGG** | **0.0116** | **0.0389** | **0.121** |
| **Likelihood ratio test: chi-square = 15.7** |  |  |  |
| **df = 8** |  |  |  |
| **Omnibus p = 0.0471** |  |  |  |
| **HAPLOTYPES: Active TB vs. LTBI** | **FREQ** | **P: Haplotype-specific test** | **P: After controlling specific haplotypes** |
| **TGATGGGAG** | **0.0167** | **0.00538** | **0.00353** |
| **TGACTTTGG** | **0.0121** | **0.013** | **0.00195** |
| **TGATTTGGG** | **0.0211** | **0.0317** | **0.00108** |
| **TGATTGGGG** | **0.0113** | **0.032** | **0.00108** |
| **TGATGGGGG** | **0.61** | **0.0357** | **0.001** |
| **Likelihood ratio test: chi-square = 32.3** |  |  |  |
| **df = 10** |  |  |  |
| **Omnibus p = 0.000359** |  |  |  |
| **LTBI vs. No LTBI** | **FREQ** | **P: Haplotype-specific test** | **P: After controlling specific haplotypes** |
| **Likelihood ratio test: chi-square = 13** |  |  |  |
| **df = 7** |  |  |  |
| **Omnibus p = 0.0725** |  |  |  |
